# The American Heartworm Society and Association of Shelter Veterinarians’ 2024 Shelter Heartworm Management Practices Survey

**DOI:** 10.1186/s13071-026-07502-1

**Published:** 2026-07-06

**Authors:** Juliana Xue, Elizabeth Fuller, Brian DiGangi, Uri Donnett

**Affiliations:** 1 RSPCA Victoria, Melbourne, Victoria Australia; 2 Charleston Animal Society, Charleston, SC USA; 3 EveryPet, Jacksonville, FL USA; 4 Dane County Humane Society, Madison, WI USA

**Keywords:** Animal shelter, Canine heartworm, Feline heartworm, Ferret heartworm, Heartworm management, *Dirofilaria immitis*

## Abstract

**Background:**

The 2024 Shelter Heartworm Management Practices Survey was performed to update current knowledge on heartworm management practices in animal shelters, including prevention, diagnosis, and treatment.

**Methods:**

The survey mirrored the 2019 survey with updates to questions based on new products and practices. The electronic survey was then distributed to veterinarians working with animal shelters in North America.

**Results:**

A total of 135 responses were received and distributed across private humane societies, municipal shelters, traditional shelters, breed/species-specific rescues, and foster-based organizations. Most organizations (77%) provided monthly heartworm preventives for dogs, using oral ivermectin products per label (50%), followed by oral milbemycin (33%). Shelters in the Midwest provided the highest percentage of heartworm preventives to both dogs and cats (37% and 40%), followed by the Southeast (31% and 34%). Heartworm testing was conducted in 76.2% of shelters for all dogs > 6 months of age, primarily using antigen testing (96.4%). Heartworm treatment was provided to all infected dogs in 66.1% of shelters. Those that only treated some dogs (22.9%) made treatment decisions based on adoptability (48.1%) and resources (25.9%). Primary adulticidal treatment was a three-dose melarsomine protocol (70.5%), followed by a two-dose melarsomine protocol (22.9%). A majority included doxycycline or minocycline and other common adjunctive treatments included steroids (75.3%) and macrocyclic lactones (60.2%). Heartworm-positive dogs were typically housed in the shelter (67%) or foster homes (49.5%) and made available for adoption. Exercise restrictions were applied in 80.6% of shelters, starting at diagnosis (55.8%) and continuing for several weeks after the last melarsomine injection (89.6%). During the restriction period, 58.2% of the shelters used psychopharmaceutical medications. Heartworm prevention was provided by 49.1% of shelters who admitted cats and 25.9% of those who admitted ferrets. Compared to 2019, shelter respondents reported increased use of preventives, microfilaria testing, doxycycline and three-dose melarsomine treatment for dogs, and a decrease in use of protocols for prevention of feline heartworm disease.

**Conclusions:**

Survey results highlight the challenges shelters have faced and the improvements made over the past 5 years, offering insights for targeted operational and educational improvements.

**Graphical Abstract:**

**Figure:**
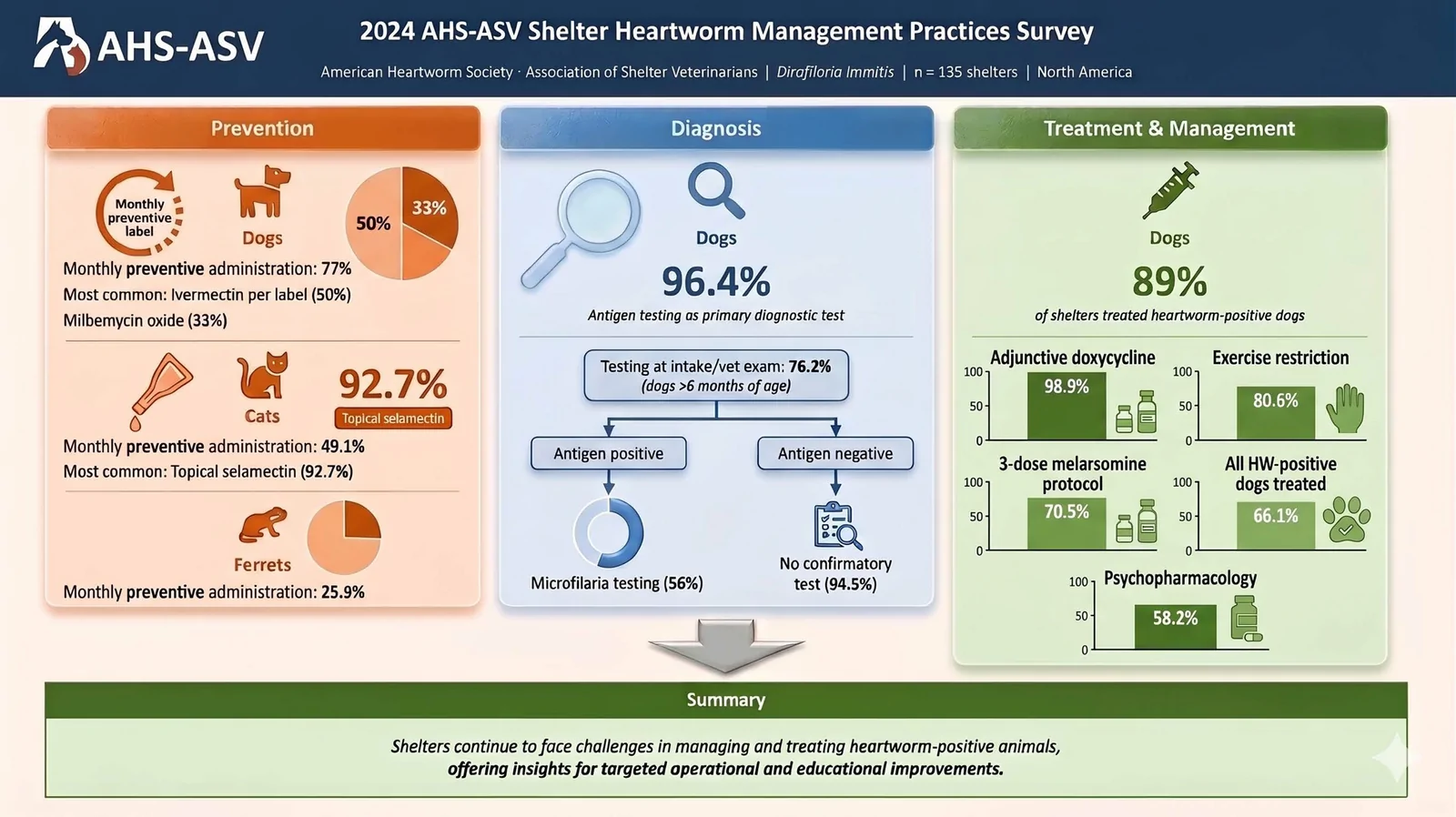

## Background

Heartworm disease, caused by *Dirofilaria immitis*, is a globally expanding vector-borne disease and the parasite has been identified in more than 20 mosquito species [1]. In 2024, an estimated 5.8 million dogs and cats entered animal shelters and rescue organizations in the United States (U.S.) [2]. Reported heartworm prevalence in dogs in the U.S. ranges from 14.6% to 48.8%, with shelters in the southeastern U.S. demonstrating consistently higher incidence rates [3, 4]. Based on these estimates, approximately 0.84 to 2.83 million dogs entering U.S. shelters annually may be infected with heartworms. Shelters with limited resources are more likely to euthanize or release heartworm-positive dogs and cats without treatment [5, 7]. Given the clinical significance of heartworm disease and its potential for geographic spread, raising awareness is essential to protecting both sheltered and owned pets worldwide from this preventable and treatable disease. The American Heartworm Society (AHS) continues to collaborate with the Association of Shelter Veterinarians (ASV) to provide support and advance the practice of shelter medicine regarding the management of heartworm disease in shelter animals. To date, a total of three surveys on heartworm management practices in North American animal shelters have been conducted [6, 7].

Previous surveys were performed in 2014 and 2019. Results of these previous surveys highlighted challenges reported by shelters, particularly regarding treatment costs, adopter and client education, foster home management, and the extended length of stay for heartworm-positive animals [6, 7]. The results of the 2014 survey aided in the development of the ASV Heartworm Disease Task Force in 2015, which developed informational documents on canine and feline heartworm disease and a series of educational brochures for distribution to adopters [8]. The results of the 2019 survey aided in the development of the ASV–AHS working group in 2020, which developed the Decision-Making Considerations for Heartworm Management in Shelter Dog Populations tool [9].

The American Veterinary Medical Association has also reported a concerning increase in heartworm incidence despite preventive efforts across the U.S. [10]. The increase in heartworm incidence is likely multifactorial. Contributing factors include low owner compliance, with only one-third of dogs reportedly receiving a single dose or more of heartworm prevention; the travel and relocation of heartworm-positive dog and changing weather patterns that promote mosquito proliferation [1, 3, 11].

In response to these growing challenges and the concerning rise in heartworm-positive cases across the U.S., the AHS and ASV conducted a repeat survey as a collaborative effort to understand current practices in management, diagnosis, and treatment of heartworm disease in animal shelters in North America. This manuscript seeks to report the findings from the 2024 survey and identify ongoing educational and operational gaps that may hinder effective heartworm disease prevention and management in shelters.

## Methods

### Survey design

The initial survey questions were a base set of 53 questions used for the 2019 survey. Questions were reviewed and edited by the ASV–AHS working group to ensure that they included all relevant and currently available products. Additional questions were added to clarify practices and address gaps in previous survey questions. The final 2024 survey comprised a total of 60 questions. Once finalized, the bank of questions was reviewed by both the AHS and ASV boards and tested by a small group of shelter practitioners. The final survey was then performed using an online survey tool (SurveyMonkey^Ⓡ^, San Mateo, CA). The target audience for the survey was shelter veterinarians across North America. The survey was disseminated via multiple electronic mailing services, social media shelter veterinary groups, and state shelter federated associations. Participants who completed the survey were eligible for inclusion in a drawing for a monetary grant reward. Participants could choose to complete the survey anonymously or to disclose their identity and/or organization.

### 2024 analysis

The survey questions were reviewed and analyzed using descriptive statistical methods using Microsoft Excel. Open-ended and free-text responses were examined individually and systematically categorized according to emergent themes. The resulting classifications were subsequently cross validated among the authors.

### Comparison analysis

The list of all participants who disclosed their identity and organization was cross-referenced with those who did so for the 2019 survey. For all organizations that completed both surveys, the answers from 2019 were compared with those from 2024 using descriptive statistical methods. McNemar’s chi-square test was performed to compare the change in proportions between the responses each year using MedCalc software. Statistical significance was set at *p* < 0.05.

## Results

### Demographics

A total of 135 responses were received in the 2024 survey. The majority of the respondents were from private humane societies/SPCA organizations (*n* = 50, 49%), followed by municipal shelters (*n* = 28, 27.5%) and traditional (public–private partnership) shelters (*n* = 19, 18.6%). The lowest response rates were from breed/species-specific rescues (*n* = 2, 2%) and foster-based organizations (*n* = 1, 1%). Respondents were relatively evenly distributed across the Midwestern (30.4%), Southeastern (29.4%) and Western (26.5%) regions of the U.S., with the fewest respondents from the Northern region (14.7%). Respondent shelters reported a median annual intake of 1303 dogs and 1600 cats. The median prevalence of dogs testing positive for heartworm disease was 1%, with the highest prevalence (70%) reported by a shelter in the Southeastern region. Among shelters who admitted and performed heartworm testing in cats, the median prevalence was 0%, with the highest prevalence (10%) also originating from the same Southeastern shelter reporting the highest canine heartworm-positive results (Table 1).

**Table 1:** Demographic characteristics of 135 respondent shelters

Characteristic	No. of responses	Response	*N* or *N* (%)
Previous year canine intake	96	Min	2
		Max	20,000
		Median	1303
Approx. percentage heartworm-positive dogs	74	Min	0
		Max	70
		Median	1
Previous year feline intake	96	Min	50
		Max	15,000
		Median	1600
Approx. percentage heartworm-positive cats	29	Min	0
		Max	10
		Median	0
Type of organization	102	Private humane society or SPCA	50 (49.0)
		Municipal animal control facility	28 (27.5)
		Traditional shelter (Animal control, private humane society/SPCA)	19 (18.6)
		Foster-based	1 (1.0)
		Sanctuary	0
		Breed/species-specific rescue	2 (2.0)
Graphic location	102	Southeast US	30 (29.4)
		Midwest US	31 (30.4)
		Northeast US	15 (14.7)
		Western US	27 (26.5)
		Other	Hawaii (0.01)
Annual medical budget	23	Min	60,000
		Max	7,000,000
		Median	200,000
How many veterinarians are employed to manage the shelter population?	102	Full time	88 (86.3)
		Part time	43 (42.2)
		Contracted	40 (39.2)

### Canine results

The majority of the respondents admitted dogs (*n* = 130, 96.3%). Of these, 105 respondents (77.8%) reported having some form of standard operating procedures (SOPs) in place for managing canine heartworm disease. These SOPs primarily addressed diagnostic testing and treatment (*n* = 100, 97.1%), prevention (*n* = 94, 91.3%), and adoption counseling (*n* = 69, 67.0%). Most shelters (*n* = 96, 77.4%) reported providing monthly heartworm preventives for dogs. The most commonly used product was oral ivermectin (used per label) (*n* = 47, 50%), followed by oral milbemycin (*n* = 31, 33.0%), topical selamectin (*n* = 26, 27.7%), oral moxidectin (*n* = 25, 26.6%), topical moxidectin (*n* = 18, 19.2%), oral ivermectin (used extra-label) (*n* = 6, 6.4%), and finally, parenteral moxidectin (*n* = 5, 5.3%).

Most shelters performed heartworm testing in dogs > 6 months of age (*n* = 93, 76.2%), typically at shelter intake and/or during veterinary examinations (*n* = 77, 68.8%). Only 8.2% of the shelters did not test for heartworm disease in dogs. Antigen testing was the primary diagnostic method reported (*n* = 107, 96.4%). Fewer respondents indicated using microfilaria testing as a screening tool (*n* = 32, 28.8%). A small number of responses indicated that serum antibody testing was used in dogs (*n* = 7, 6.3%). Of the 109 respondents who answered the question regarding secondary diagnostic testing, 27.5% (*n* = 30) reported that no secondary diagnostic test would be used. In contrast, 72.5% indicated that some form of secondary diagnostic testing would be performed following a positive antigen test. When respondents were asked about their preferred secondary diagnostic test, 38.5% (*n* = 42) selected repeated antigen testing, 9.2% (*n* = 10) selected heat-treated antigen testing, and 56% (*n* = 61) selected microfilaria testing. When microfilaria testing was employed, most respondents used a drop of blood and observed for motility (*n* = 43, 39.5%), a Modified Knott’s test conducted by a reference laboratory (*n* = 26, 23.9%), or a blood smear (*n* = 23, 21.1%). The majority of shelters did not perform secondary testing if the primary test was negative (*n* = 103, 94.5%).

Approximately two-thirds of the shelters reported treating all heartworm-positive dogs (*n* = 78, 66.1%). Others made treatment decisions based on shelter- and dog-related factors (*n* = 27, 22.9%). Factors which influenced treatment decisions were the adoptability of the animal (other behavioral and medical comorbidities) (*n* = 13, 50.0%), or the resources of the shelter (financial, space, foster home availability) (*n* = 8, 30.8%). The most commonly used adulticide treatment was a three-dose melarsomine protocol (*n* = 74, 70.5%), followed by a two-dose melarsomine protocol (*n* = 24, 22.9%). Adjunctive doxycycline or minocycline was used by most shelters employing either the three-dose (97.3%) or two-dose (87.5%) melarsomine protocols. All shelters that reported using a non-arsenical protocol included doxycycline or minocycline in that protocol, with 96.9% of respondents administering these medications for 4 weeks.

Dogs were sedated or under general anesthesia during melarsomine administration in almost half of the shelters (*n* = 46, 49.5%). Heartworm-positive dogs were typically housed in the shelter (*n* = 69, 67%) or placed in foster homes and made available for adoption (*n* = 51, 49.5%). Preferences for the timing of spay-neuter surgery in relation to heartworm treatment varied among shelters. The most common approach was to perform surgery immediately after diagnosis but before treatment initiation (*n* = 26, 28.9%), followed by after starting doxycycline therapy (*n* = 20, 22.2%), and prior to heartworm diagnosis (*n* = 18, 20%). Fewer shelters preferred performing surgery after completing melarsomine injections (*n* = 12, 13.3%) or performed surgery on the day of melarsomine injection (*n* = 4, 4.4%). Only a small proportion (*n* = 4, 4.4%) reported that the timing of surgery was not a concern in heartworm-positive dogs.

Over half of the shelters reported administering psychopharmaceuticals to heartworm-positive dogs during treatment (*n* = 60, 58.3%). The most commonly used medications were trazodone (*n* = 60, 100%) and gabapentin (*n* = 55, 91.7%). Most shelters reported enforcing exercise restriction for heartworm-positive dogs at all times (*n* = 83, 80.6%) or sometimes (*n* = 6, 5.8%) during treatment. Over half implemented restrictions at the time of diagnosis (*n* = 53, 55.8%), while the remainder began after treatment initiation (*n* = 42, 44.2%).

The most frequently cited challenges in managing canine heartworm disease included cost (*n* = 24, 22.4%), treatment-related factors such as expense, compliance, and duration (*n* = 22, 20.6%), prolonged length of stay (*n* = 17, 15.9%), and balancing exercise restriction with the maintenance of positive mental welfare during prolonged confinement (*n* = 15, 14%) (Table 2).

**Table 2:** Canine heartworm disease prevention, diagnosis, and management practices in 130 canine shelters

Question	No. of responses	Response	*N* or *N* (%)
Does your shelter take in dogs?	135	Yes	130 (96.3)
		No	5 (3.7)
Do you have a standard operating procedure (SOP) for canine heartworm disease management?	126	Yes	105 (77.8)
		No	21 (15.6)
Which of the following topics are covered by your SOP?	103	Prevention	94 (91.3)
		Diagnosis	100 (97.1)
		Treatment	100 (97.1)
		Adoption	69 (67.0)
Do dogs receive monthly heartworm preventative?	124	Yes	96 (77.4)
		No	28 (22.6)
What preventive agent(s) are used most commonly?	94	Oral ivermectin (per label)	47 (50.0)
		Oral ivermectin (extra-label)	6 (6.4)
		Oral milbemycin	31 (33.0)
		Topical moxidectin	18 (19.2)
		Oral moxidectin	25 (26.6)
		Topical selamectin	26 (27.7)
		Parenteral moxidectin	5 (5.3)
Are dogs tested for heartworm infection?	122	Some dogs are tested	19 (15.6)
		All dogs > 6 months of age are tested	93 (76.2)
		No	10 (8.2)
When dogs are initially tested?	112	Prior to admission	3 (2.8)
		During intake and/or veterinary examination	77 (68.8)
		During spay/neuter surgery	21 (18.8)
		During the transfer/transport out process/CVI exam	4 (3.6)
		During the adoption process	5 (4.5)
		Other (please specify)	2 (1.8)
What type of screening test(s) is/are used? (Select all that apply.)	111	Microfilaria	32 (28.8)
		Antigen	107 (96.4)
		Serum antibody	7 (6.3)
Which secondary diagnostic tests are typically performed after a positive screening test? (select all that apply)	109	Antigen testing	42 (38.5)
		Heat-treated antigen testing	10 (9.2)
		Microfilarial testing	61 (56.0)
		Complete blood count	32 (29.4)
		Blood chemistry analysis	32 (29.4)
		Urinalysis	9 (8.3)
		Thoracic radiographs	33 (30.3)
		Echocardiogram	1 (0.9)
If Microfilaria testing is performed which type of test is used? (Select all that apply.)	97	Drop of blood observed for motility	43 (39.5)
		Blood smear	23 (21.1)
		Hematocrit tube	3 (2.8)
		Modified Knotts—in house	2 (1.8)
		Modified Knotts—reference lab	26 (23.9)
Are secondary diagnostic tests pursued following a negative screening test?	109	Yes	6 (5.5)
		No	103 (94.5)
Are heartworm-positive dogs treated by your organization?	118	No, positive dogs are not treated	13 (11.0)
		All positive dogs are treated	78 (66.1)
		Some positive dogs are treated. (post adoption, rescue/adoption placement)	27 (22.9)*
Some positive dogs are treated. (please specify)	26*	Treated post adoption	5 (19.2)
		Adoptability of the animal (Behavioral and Medical)	13 (50.0)
		Resources of the shelter (Financial, space, foster home availabilities)	8 (30.8)
		Others (N/a)—excluded	1
How are heartworm-positive dogs managed?	9	Dogs are transferred to another facility for treatment	5 (55.6)
		Dogs are placed for adoption without treatment	2 (22.2)
		Dogs are euthanized	3 (33.3)
		Transfer to rescue	3 (33.3)
What is the primary adulticide protocol?	105	Melarsomine dihydrochloride two-injection protocol (2 injections, 24 h apart)	24 (22.9)
		Melarsomine dihydrochloride three-injection alternate protocol (1 injection, followed 1 month later by 2 injections, 24 h apart)	74 (70.5)
		Moxidectin (topical) + doxycycline	3 (2.9)
		Moxidectin (injectable) + doxycycline/minocycline	2 (1.9)
		Ivermectin/moxidectin + doxycycline/minocycline	0
		Ivermectin/moxidectin monotherapy	0
Are dogs routinely sedated or anesthetized during injection administration?	93	Yes	46 (49.5)
		No	47 (50.5)
When is spay-neuter surgery performed in relation to adulticidal therapy? (Choose the best answer.)	90	Prior to heartworm diagnosis	18 (20)
		Immediately after diagnosis, prior to initiating treatment protocol	26 (28.9)
		After beginning doxycycline therapy	20 (22.2)
		After completing doxycycline therapy	6 (6.7)
		On the day of the melarsomine injection	4 (4.4)
		After melarsomine injections	12(13.3)
		Other (Timing does not matter)	4 (4.4)
What adjunctive treatments are typically utilized?	93	Macrocyclic lactones	56 (60.2)
		Doxycycline or minocycline	92 (98.9)
		Steroids	70 (75.3)
		None	1 (1.1)
		Other (pain relief—opioids, NSAIDs, gabapentin)	11 (11.8)
Where are dogs housed after being identified as heartworm positive?	103	In shelter, unavailable for adoption	29 (28.2)
		In shelter, available for adoption	69 (67.0)
		Foster home, available for adoption	51 (49.5)
		Foster home, unavailable for adoption	29 (28.2)
		Adopted home	39 (37.9)
		Other (Foster to Adopt)	3 (2.9)
Are anxiolytics, antidepressants, or other psychopharmaceuticals routinely utilized during the period of exercise restriction—specifically for heartworm treatment purposes and not for other behavioral reasons?	103	Yes, if in shelter	8 (7.8)
		Yes, if in foster home	3 (2.9)
		Yes, in shelter and foster home	49 (47.6)
		No	43 (41.8)
If yes, which drugs are used most frequently? (Select all that apply.)	60	Trazodone	60 (100)
		Gabapentin	55 (91.7)
		Acepromazine	5 (8.3)
		Clonidine	10 (16.7)
		SSRIs (e.g., fluoxetine)	4 (6.7)
		TCAs (e.g., clomipramine)	1 (1.7)
		Benzodiazepines (e.g., alprazolam)	0 (0)
Do you exercise restrict heartworm-positive dogs?	103	No	7 (6.8)
		Yes, all the time	83 (80.6)
		Yes, sometimes (pending on mental health status of the animal)	6 (5.8)
When do you start exercise restriction?	95	At the time of diagnosis	53 (55.8)
		After treatment is initiated	42 (44.2)
How long do you restrict exercise?	96	Until last melarsomine injection	1 (1.0)
		Until several weeks after last melarsomine injection	86 (89.6)
		Until antigen negative	6 (6.3)
What is your shelter’s greatest challenge with canine heartworm management?	107	Cost	24 (22.4)
		Adopter/client education	4 (3.7)
		Foster home management	7 (6.5)
		Length of stay	17 (15.9)
		Activity restriction and behavioral management	15 (14.0)
		Treatment (cost, time, compliance of treatment process)	22 (20.6)
		Diagnosis	5 (4.7)
		Volume of cases	3 (2.8)
		Prevention	6 (5.6)
		Other	4 (3.7)

^*^One respondent did not provide reason of why some dogs may or may not receive treatment; therefore, the total respondent was reduced from 27 to 26 in the following question

### Feline results

A total of 113 respondent shelters admitted cats (95%), and most did not have SOPs for managing heartworm disease (*n* = 96, 85%). Among the 15% of shelters with SOPs, these primarily addressed prevention (*n* = 11, 64.7%), diagnostic testing (*n* = 10, 58.8%), and treatment (*n* = 7, 41.2%). Approximately half of the shelters routinely administered monthly heartworm preventives to cats (*n* = 55, 49.1%). The most commonly used product was topical selamectin (*n* = 51, 92.7%), followed by oral moxidectin (*n* = 10, 18.2%) and parenteral moxidectin (*n* = 2, 3.6%). The majority of shelters reported that cats were not routinely tested for heartworm infection (*n* = 82, 73.2%). When testing was performed, it most commonly occurred during intake examinations (*n* = 12, 41.4%), followed by testing at the time of veterinary examinations (*n* = 11, 37.9%), or spay/neuter surgeries (*n* = 11, 37.9%). Antigen testing was the preferred screening method (*n* = 22, 75.9%), followed by serum antibody testing (*n* = 8, 27.6%). Upon a positive screening test result, some shelters conducted secondary diagnostic tests (*n* = 12, 41.1%), most frequently heat-treated antigen testing (*n* = 9, 50.0%), use of an alternative antigen test (*n* = 8, 44.4%), or microfilaria testing (*n* = 8, 44.4%). Nearly half of the respondents stated heartworm-positive cats were made available for adoption (*n* = 15, 46.9%), while only two of the 32 respondent shelters indicated that heartworm-positive cats would be euthanized (1.1%). Nearly two-thirds of the respondent shelters did not treat heartworm-positive cats (*n* = 23, 62.2%). Among those providing treatment, the most common approach was monthly administration of a macrocyclic lactone (*n* = 13, 35.1%). The major challenge reported in managing feline heartworm disease was diagnostic testing, largely due to the absence of routine screening tools or tests and ability to interpret them and act on diagnosis (*n* = 25, 26.3%) (Table 3).

**Table 3:** Feline heartworm disease prevention, diagnosis and management practices in 113 feline shelters

Question	No. of responses	Response	*N* or *N* (%)
Does your shelter take in cats?	119	Yes	113 (95.0)
		No	6 (5.0)
Do you have a standard operating procedure (SOP) for feline heartworm disease management?	113	Yes	17 (15.0)
		No	96 (85.0)
Which of the following topics are covered by your SOP?	103	Prevention	11 (64.7)
		Diagnosis	10 (58.8)
		Treatment	7 (41.2)
		Adoption	5 (29.4)
Do cats receive monthly heartworm prevention?	112	Yes	55 (49.1)
		No	57 (50.89)
What preventive agent(s) are used most commonly?	55	Oral ivermectin (per label)	0
		Oral ivermectin (extra-label)	0
		Oral milbemycin	0
		Topical moxidectin	0
		Oral moxidectin	10 (18.2)
		Topical selamectin	51 (92.7)
		Parenteral moxidectin	2 (3.6)
Are cats tested for heartworm infection?	112	Some cats are tested	16 (14.3)
		All cats > 6 months of age are tested	14 (12.5)
		No	82 (73.2)
When cats are initially tested?	39	Prior to admission	1 (3.5)
		During intake examination	12 (41.4)
		During veterinary examination	11 (37.9)
		During spay/neuter surgery	11 (37.9)
		During the transfer/transport out process/CVI exam	2 (6.9)
		During the adoption process	1 (3.5)
		Other (if cats show clinical signs)	6 (20.7)
What type of screening test(s) is/are used?	28	Microfilaria	0
		Antigen	22 (75.9)
		Serum antibody	8 (27.6)
		Other	0
Are secondary diagnostic tests pursued following a positive screening test?	27	Yes	12 (41.1)
		No	11 (37.3)
		Sometimes	4 (14.8)
Which secondary diagnostic tests are typically performed after a positive screening test?	16	Antigen testing	8 (44.4)
		Heat-treated antigen testing	9 (50)
		Microfilarial testing	8 (44.4)
		Complete blood count	5 (27.8)
		Blood chemistry analysis	4 (22.2)
		Urinalysis	1 (5.6)
		Thoracic radiographs	6 (33.3)
		Echocardiogram	0
How are heartworm-positive cats managed?	32	Cats are placed for adoption	15 (46.9)
		Cats are transferred to another facility	0 (0)
		Cats are euthanized	2 (1.1)
		Other (please specify)	15 (46.9)
What management modalities are typically utilized in heartworm-positive cats?	37	Not applicable	23 (62.2)
		Monthly macrocyclic lactone administration	13 (35.1)
		Doxycycline or minocycline	4 (10.8)
		Corticosteroids	4 (10.8)
		Other (please specify)	0
What is your shelter’s greatest challenge with feline heartworm management?	95	Cost	6 (6.3)
		Adopter/client education	4 (4.2)
		Foster home management	0
		Length of stay (getting them adopted out)	4 (4.2)
		Activity restriction	0
		Treatment	9 (9.5)
		Diagnosis (cats do not get screened for HW)	25 (26.3)
		Volume of cases	0
		Prevention	2 (2.1)
		Other (the pathway options for these cats)	2 (2.1)

### Ferret results

Half of the respondents admitted ferrets (*n* = 54, 51.0%), and among these, only 25.9% reported using monthly heartworm preventives.

### 2019–2024 Comparison

Of the 135 total respondents in 2024 and the 242 total respondents in 2019, only 19 organizations completed surveys both years and identified themselves both years. Responses from these 19 organizations were included in the comparison. The majority of these organizations were private non-profits (68.4%) with the remainder being either municipal or non-profits with a contract (both 15.7%). Respondents were relatively evenly distributed across the Midwestern (37%, *n* = 7), Southeastern (26%, *n* = 5) and Western (26%, *n* = 5) regions of the U.S., with no respondents from the Southwestern region. The average heartworm prevalence reported by these organizations was 4.4% in 2019 and 5.5% in 2025.

Comparison showed a 10% increase in shelters providing heartworm prevention to dogs going from 16 organizations giving all dogs preventives in 2019 to 18 in 2024. This increase was statistically significant (*P* < 0.05). In both survey years, the most commonly used product was on-label ivermectin. Between 2019 and 2024, there were increases in the use of oral milbemycin, oral moxidectin, topical selamectin, and parenteral moxidectin. There was a 31.5% decrease in the number of shelters using topical moxidectin, with 7 shelters using it in 2019 and only 1 shelter reporting use in 2024 (*P* < 0.05). In 2019, 74% (14/19) of organizations reported testing all dogs vs. 79% (15/19) in 2024. This increase was statistically significant (*P* < 0.05). The initial screening test most commonly used was antigen testing in both 2019 and 2024 (17/19 and 19/19, respectively) (*P* < 0.05). For positive screening results, in 2019, 11 shelters reported using antigen tests for confirmatory testing, this decreased to 8 shelters in 2024 (*P* = 1.0). Conversely, 11 shelters reported using microfilaria testing for confirmatory testing in 2019; this number increased to 13 shelters in 2024 (*P* = 0.38). In both survey years, 3 shelters reported performing no confirmatory testing for positive samples with two shelters reporting no confirmatory testing both in 2019 and 2024.

In 2019, 79% (15/19) reported treating all positive dogs for heartworm disease vs. 89% (17/19) in 2024. This increase was statistically significant (*P* < 0.05). In 2019, 84% (16/19) reported always using doxycycline during treatment. This increased to 100% of organizations using doxycycline in 2024 (*P* < 0.05). In 2019, 1 shelter reported using both two- and three-injection protocols. The three-dose melarsomine protocol remained the most commonly used treatment option in both survey years. In 2019, the equivalent of 10 shelters reported using the three-dose protocol; this increased to 12 shelters in 2024 (*P* = 0.66). One shelter in 2019 reported selecting either a two-dose or three-dose melarsomine protocol based on patient temperament and tolerance, in 2024 they only reported using a three-dose melarsomine protocol. Use of the two-dose melarsomine protocol was reported by 6 shelters in 2019 and decreased slightly to 5 shelters in 2024 (*P* = 0.09).

Two shelters reported using oral moxidectin with doxycycline in 2019, but none reported using this protocol in 2024 (*P* < 0.05). One shelter did report using injectable moxidectin plus doxycycline in 2024. A 32% increase was observed in the proportion of shelters keeping dogs available for adoption during the treatment period, with the number of shelters increasing from 10 in 2019 to 16 in 2024 (*P* = 0.22). In 2019, only 26% (5/19) shelters used psychopharmaceuticals during the treatment period, whereas this increased to 36% (7/19) in 2024 (Table 4). This increase was statistically significant (*P* = 0.18).

**Table 4:** Comparison of shelters completing both 2019 and 2024 surveys (*n* = 19)

Question	Response	2019	2024
		*N* (%)	*N* (%)
Do dogs receive monthly heartworm preventative?	Yes	16 (84)	18 (95)
	No	3 (16)	1 (5)
What preventive agent(s) are used most commonly?	Oral ivermectin (per label)	9 (47)	9 (47)
	Oral ivermectin (extra-label)	2 (11)	1 (5)
	Oral milbemycin	5 (26)	6 (32)
	Topical moxidectin	7 (37)	1 (5)
	Oral moxidectin	N/A	2 (11)
	Topical selamectin	1 (5)	2 (11)
	Parenteral moxidectin	0	2 (11)
Are dogs tested for heartworm infection?	All dogs > 6 months are tested	14 (74)	15 (79)
	Some dogs are tested	5 (26)	4 (21)
What type of screening test(s) is/are used? (select all that apply)	Microfilaria	8 (42)	6 (32)
	Antigen	17 (89)	19 (100)
	Serum antibody	0	2 (11)
Which secondary diagnostic tests are typically performed? (select all that apply)	Antigen test	11 (58)	8 (42)
	Heat-treated antigen	2 (11)	3 (16)
	Microfilaria test	11 (58)	13 (68)
	CBC	8 (42)	6 (32)
	Chemistry	8 (42)	6 (32)
	Urinalysis	0	0
	None	3 (16)	3 (16)
Are heartworm-positive dogs treated by your organization?	All positive dogs are treated	15 (79)	17 (89)
	Some positive dogs are treated	5 (26)	2 (11)
What is the primary adulticide treatment protocol?	Melarsomine 3 injections	10.5 (55)	13 (68)
	Melarsomine 2 injections	6.5 (34)	5 (26)
	Topical Moxidectin + doxycycline	2 (11)	0
	Injectable Moxidectin + doxycycline	0	1 (5)
How often is doxycycline or minocycline used?	Always	16 (84)	19 (100)
	Sometimes	0	0
	Never	1 (5)	0
	No response	3 (16)	0
Are anxiolytics, antidepressants, or other psychopharmaceuticals routinely utilized during the period of exercise restriction—specifically for heartworm treatment purposes and not for other behavioral reasons?	Yes, in shelter	0	1 (5)
	Yes, in foster home	0	0
	Yes, in shelter and foster home	5 (26)	6 (32)
	No	14 (74)	12 (63)

N/A = Response was not an option present in that year’s version of the survey

Eighteen of the 19 organizations reported feline intake. Among shelters admitting cats, 28% (5/18) reported having a SOP for feline heartworm disease in 2019; however, this proportion decreased to 11% (2/18) in 2024. This decrease was statistically significant (*P* < 0.05). Only 21% (4/18) of shelters reported administering heartworm prevention to cats in both survey years. In 2019, six organizations reported testing some or all cats for heartworm disease, which declined to four organizations in 2024 (*P* = 0.07). Among those conducting testing, heartworm testing was reported to be incidental, as the heartworm antigen test is often included in the point of care feline FIV/FeLV tests. In 2019, four organizations reported euthanasia as the outcome for heartworm-positive cats, whereas no organizations reported this outcome in 2024 (*P* < 0.05). However, the prevailing survey text response was that testing was not done, so positive cats could not be identified.

## Discussion

Year after year incidence maps show an increased geographic distribution of heartworm disease in dogs. Since the previous survey in 2019, several major events had significant impacts on animal sheltering. The first of these was the COVID pandemic in 2020, which saw many shelters dramatically reduce their populations, place many animals in foster care, and delaying surgeries and treatments to preserve personal protective equipment. Some shelters continued to experience the long-lasting effects of these temporary practice changes as late as 2024. In 2022, the ASV also released updated Guidelines for Standards of Care in Animal Shelters, which included recommendations related to heartworm disease within the medical and transport sections [12]. Despite improvements in knowledge base and practices of shelter medicine and heartworm disease management, heartworm disease remains an important and challenging disease to manage in a shelter setting.

Amid growing concerns about the increasing incidence of heartworm across the U.S., results from the 2024 survey indicated that the majority of shelters continue to provide heartworm prevention to a majority of dogs in their care. The direct comparison of shelters from 2019 to 2024 showed an increase in preventive use. While many new broad-spectrum products have entered the market, both 2019 and 2024 surveys found that the most commonly used product type is oral ivermectin. This is likely primarily driven by cost. Interestingly, if the focus is only on the active ingredient used and not the route, 51.5% of shelters report using a moxidectin product, while 56.4% report using an ivermectin product. It appears that moxidectin may in the future overtake ivermectin as the preventive drug of choice in the shelter environment. This is likely due to the increased attention that moxidectin has received as a heartworm preventive, microfilaricide, and adulticidal molecule [13].

Antigen testing remained the most commonly used diagnostic method for canine heartworm detection. Microfilaria testing was the preferred secondary testing method for 56% of respondents following a positive antigen result. The vast majority of the respondents (94.5%) indicated they would not pursue any secondary testing if the initial antigen result was negative. This finding is concerning given the relatively low sensitivity of antigen testing in cases of low worm burden and the potential for antigen–antibody complex formation, as well as the frequent absence of reliable heartworm prevention histories prior to shelter intake. The AHS Guidelines for the Prevention, Management, and Treatment of Heartworm Infection in Dogs do not recommend routine heat-treated antigen testing, except in cases, where initial test results are inconsistent with the animal’s clinical signs or when there is no history of preventive use in a high-risk animal from a high-risk area [8]. While shelter dogs would be considered higher risk due to a lack of preventive history, not all shelter dogs originate from or reside in regions with high heartworm prevalence. In addition, the absence of paired microfilariae and antigen testing may further contribute to missed diagnoses in these high-risk populations. Many shelter organizations still do not perform parallel antigen and microfilaria testing. Increasing educational opportunities for shelters on the value of using both diagnostic methods to improve heartworm detection may be more practical and impactful than promoting routine heat-treated testing.

Serum antibody testing was a primary diagnostic method for canines reported by a small number of respondents (*n* = 7, 6.3%). For shelters testing cats for heartworm disease, it was relied upon more commonly (*n* = 8, 27.6%). While serum antibody can be a useful diagnostic tool in certain contexts, its utility for canines is much lower than for felines. The unique nature of feline infection makes serum antibody testing potentially more valuable than for canines. In addition, a cage side serum antibody test for cats is more readily available, while a canine test is not. As a result, canine samples would typically need to be submitted to a reference laboratory or run on the unvalidated feline antibody test. One recent study comparing antigen to antibody testing in shelter dogs found that both a feline and canine antibody test had relatively low sensitivity and specificity when compared to a serum antigen test [14].

Equally concerning to note was that 27.5% (*n* = 30) of shelters reported that they did not perform a secondary diagnostic test after the initial screening test if it was positive. With many respondents being from areas of the country, where the prevalence of heartworm disease is low, and their reported yearly prevalence was low, this could mean that shelters were accepting potentially false positive results. Both responses emphasize the importance of confirmatory testing for both positive and negative test results.

While this and previous surveys asked about the timing of testing, it is important to note that some shelters may have received dogs that were already tested prior to intake. The survey did not capture whether retesting occurred or whether testing protocols differed for dogs transported into respondent shelters from geographic regions with differing prevalence. While dogs are often being transported from areas of the U.S. with higher heartworm prevalence, protocols may differ for management of these dogs, and it is often done intentionally, with prior knowledge of heartworm status, with pre-treatment to reduce transmissibility, and with the express purpose of completing treatment at the destination. These practices represent areas for further exploration in future surveys.

The 2024 survey asked additional questions about microfilaria testing that were not included in the 2019 survey. Shelters that performed microfilaria testing predominantly reported that they performed an in-house test (65.2%). The AHS guidelines recommend testing in parallel with an antigen test and the Modified Knott test [15]. In this survey only 25.7% of shelters performed a Modified Knott test and the majority were sent to a reference laboratory. While the in-house tests most shelters reported using (blood smear and motility testing) have a lower sensitivity than a Modified Knott test, they are inexpensive, quick to perform, and require minimal observer training [16]. In order to increase the performance of the Modified Knott test in the shelter setting, established protocols should be implemented with training and instructions provided, and safer alternatives to formalin should be used [17].

The majority of shelters who diagnosed heartworm disease reported providing treatment (66.1%). For organizations that did not treat any heartworm-positive dogs, more organizations reported transferring the dog to another facility or rescue for treatment rather than euthanizing due to positive test. Although there has been increased awareness and publications related to non-arsenical adulticide protocols, the 2024 survey still found the majority of shelters that treat heartworm disease were using a melarsomine-based protocol. In addition, nearly all respondents (98.9%) indicated they used doxycycline or minocycline as adjunctive therapy with the three-dose melarsomine protocol. While it appears that most shelters surveyed follow the AHS treatment guidelines, the survey did not ask specifics of doxycycline dosage, duration, or timing between doxycycline and melarsomine injections. Recent studies have shown that dogs can have positive clinical outcomes with shorter durations of doxycycline or lower doses and it is possible that shelters have adopted these protocols to reduce length of stay and optimize limited shelter resources [18].

Nearly half of the respondents in the 2024 survey (49.5%) reported using sedation or general anesthesia during melarsomine administration. Most respondents also reported performing spay-neuter surgery prior to diagnosis and adulticidal treatment (82.2%). The use of a cardiovascular sparing anesthetic protocol has been shown to result in no perioperative or postoperative complications in heartworm-positive dogs exhibiting no or mild clinical signs [19]. These dogs were categorized as American Society of Anesthesiologists (ASA) status class I or II [20]. In contrast, dogs with moderate to severe clinical signs, particularly those affected by pulmonary hypertension, pulmonary thromboembolism, or caval syndrome, would require general anesthesia with extreme caution. In one report, four heartworm-positive dogs with caval syndrome underwent general anesthesia for surgical worm extraction, and all four patients recovered uneventfully [20]. Newer research has reported positive outcomes for dogs undergoing spay-neuter surgeries at a high-quality high-volume clinic and in the context of prolonged anesthetic surgical times in a veterinary training program, both without alteration of standardized anesthetic and operational protocols. These reports provide further evidence that routine surgical procedures are safe to perform on asymptomatic heartworm-positive dogs prior to the start of treatment [21, 22].

The use of psychopharmaceuticals was also high in this survey population (58.2%), with trazodone and gabapentin being the most common choices. A recent publication described a heartworm treatment program in a high-volume, outpatient community clinic, where patients were given trazodone (5–10 mg/kg) orally prior to leaving home for melarsomine injection. Dogs that continued to show a high level of fear, anxiety, and stress were given additional sedatives with butorphanol and dexmedetomidine administered intramuscularly 30 min before melarsomine injection [23]. This report showed that this protocol was generally safe and well-tolerated by the heartworm-positive dogs.

Heartworm prevalence in cats in the U.S. is estimated to range between 3.5 and 15%, though the true prevalence is believed to be significantly underestimated [24, 25]. Consequently, accurate diagnosis and routine prevention are essential to prevent the spread of heartworm into non-endemic areas. Heartworm prevalence was reported to be the highest in westernmost U.S. states (5.4%) [24]. The 2024 survey identified several diagnostic challenges in cats, with the majority of shelters (73.2%) not routinely testing cats. This is likely due to the low observed prevalence of heartworm in shelters, limited treatment options, difficulties in interpreting test results for heartworm-positive cats, and the absence of established SOPs for feline heartworm management. In addition, the proportion of cats entering shelters under 6 months of age is higher than that of dogs across the U.S., so shelters may focus their efforts and SOPs on more prevalent diseases of juvenile cats [26].

A large number of shelters reported using feline heartworm prevention (49.1%) with topical selamectin being the most commonly used preventive (92.7%) in the survey results. This is likely due to cost, ease of administration, and broad-spectrum use for other parasites of concern. Antigen testing was reported as the preferred method for feline heartworm testing in this survey; however, the sensitivity of heartworm antigen testing in cats is lower than in dogs, making it a less reliable standalone diagnostic method. Instead, the AHS feline guidelines recommend using a combination of antigen and antibody testing in cats to improve the likelihood of an accurate diagnosis [27].

Ferrets are highly susceptible to heartworm disease and may develop severe clinical signs or death if infected [28]. Over half (51.4%) of 2024 respondents reported that their shelters admitted ferrets, yet only 25.9% reported providing heartworm preventives. The AHS recommends year-round heartworm prevention for ferrets, with heartworm testing before initiating prophylactic treatment in ferrets over 6 months of age [28, 29].

One notable change from the 2019 survey to the 2024 survey was the decline in organization participation. In 2019, 242 organizations took the survey, while only 135 organizations completed the survey in 2024. There were also notable shifts in the demographics of survey respondents with increases in the proportion of shelters from the Midwest and Western U.S. regions completing the survey in 2024 when compared to 2019. These changes in survey participation and demographics limit direct comparison between 2019 and 2024 results. However, limited comparison did show positive changes in management in the areas of prevention with increased use, testing using antigen tests, and the use of doxycycline with treatment. In addition, more shelters were making heartworm-positive dogs available for adoption during treatment. The decrease in participation may be due to the distribution of the survey itself, movement of veterinarians who previously took the survey out of the field or previous positions, or even due to survey exhaustion. The changes in demographic participation are likely related to the distribution and advertisement of the survey. Distribution of the survey relied on peer-to-peer marketing, online organizational promotion, and historic mailing list and participant lists. These methods may, at this point 12 years past the first survey, be outdated and require modification to encourage participation. In addition, the survey was distributed to all known state federated shelter associations for distribution. While we did not get responses from each of these organizations about whether the survey was distributed, there are only 18 states with this type of organization identified of which 8 are located in the Northeast, 3 in the West, 4 in the Midwest, and only 2 in the Southeast. The fact that the survey is only meant to be completed by a veterinarian would also limit responses from organizations that may not have a veterinarian or may contract with a veterinarian outside of shelter medicine.

## Conclusions

This study provides an updated overview of current shelter management practices for canine heartworm disease. The majority of organizations in this study provided dogs with heartworm prevention, tested for infection using antigen testing, and treated positive dogs with a melarsomine-based protocol. These interventions were implemented while balancing canine welfare, shelter capacity, length of stay, and resource allocation. Ongoing challenges and needs include financing of heartworm treatment, communicating care expectations to fosters and adopters, and identifying safe, effective, and shortened treatment protocols. Future surveys would benefit from including questions on the transport and management of known positive dogs. Heartworm disease management in shelter cats and ferrets, with an emphasis on prevention remain important, as these species appear to have taken a back seat to canines.

## Data Availability

Data supporting the main conclusions of this study are included in the manuscript.
